# Simultaneous Treatment of Pseudo-gynecomastia and Lateral Chest in Patients with Massive Weight Loss

**DOI:** 10.1007/s00266-025-04658-6

**Published:** 2025-01-21

**Authors:** Moaz Fathy Khalifa Fayez Affara, Mohamed Samir Badawy, Khaled Ahmed Reyad, Amr Mabrouk

**Affiliations:** Department of Plastic, Burn and Maxillofacial Surgery, Ain Shams University, 7 Mohsen Roshdy street, Nasr City, Cairo, 11731 Egypt

**Keywords:** Weight loss, Psuedo-gynecomastia, Infra-mammary excision, Inferior pedicle

## Abstract

**Background:**

Pseudogynecomastia in MWL patients is characterized by excess skin in chest, lateral chest, axilla and upper abdomen without enlargement of the breast glandular component. The aim of this work was to study long-term aesthetic outcomes of correction of severe pseudogynecomastia post-weight loss with inferior pedicle technique with some refinements.

**Methodology:**

This prospective study included 15 patients underwent chest contouring after massive weight loss within period of 2 years between January 2022 and January 2024. All patients were subjected to local examination (position of nipple–areola complex and degree of ptosis and chest anthropometry) and photographic assessment, and pre- and postoperative results were compared. Patient satisfaction survey was done.

**Results:**

The ages ranged from 20 to 45 years (mean 32.67 years), the previous weight ranged with mean 158.8, the mean current weight was 87.53, and the mean BMI was 28.49. The time of weight loss ranged from 8 to 48 months (mean 20.07 months), and the duration of weight stability ranged from 6 to 24 months with mean 10.0 months. Regarding the complications, no major complications required readmission, four patients had minor complications (26.7%), one (6.7%) case had asymmetry of the NAC, one (6.7%) case had minor hematoma, one (6.7%) case had seroma, and one (6.7%) case had partial wound dehiscence. The overall satisfaction rate was high.

**Conclusion:**

Patient satisfaction with male chest contouring for pseudogynecomastia following significant weight loss is excellent. In this study, we provide a reliable technique for management of pseudogynecomastia in post-MWL with good outcomes.

**Level of Evidence IV:**

This journal requires that authors assign a level of evidence to each article. For a full description of these Evidence-Based Medicine ratings, please refer to the Table of Contents or the online Instructions to Authors www.springer.com/00266.

**Supplementary Information:**

The online version contains supplementary material available at 10.1007/s00266-025-04658-6.

## Introduction

The rise in bariatric surgery has led to an increase in the demand for body contouring after massive weight loss (MWL). The percentage of patients, who have had bariatric surgeries, raised to 21.7% in 2011 in comparison with 15.4% in 2002. Male patients represent 15 to 20% of the total number of patients who had bariatric surgeries. However, this number is increasing [1]. Despite the significant costs, and the resultant skin excess, there is a high demand of bariatric surgery aiming to get better body shape and avoid fatal obesity complications. This led by default to increase the need for post- bariatric reshaping procedures [2].

Deformity of the chest wall is a part of surrounding aesthetic units, such as the upper abdomen and lateral chest. Based on the degree of ptosis, lateral chest roll extension, and upper abdominal laxity, patients were categorized into one of three categories for pseudogynecomastia [3]. Surgical techniques for management of this condition were adapted that in large pseudogynecomastia, excess skin has lost its elastic properties. Overstretch of the nipple–areola complex is also present. For these reasons, surgery must combine removal of the excess skin, reduction the size of the areola, repositioning the NAC and restore the masculine appearance of the male chest, to avoid inadequate results and the burden of a second operation [4–6].

Many surgical techniques were described for treating pseudogynecomastia, although optimizing results in patients with the most severe grades of deformity can be challenging [7]. The aim is to restore a masculine chest by resecting breast tissue and excess skin, reducing and adequately replacing the nipple–areola complex, and violating the infra-mammary fold while minimizing scarring to the skin [8]. Standard surgical treatment of excess male breast tissue began with the intra-areolar semicircular approach. Although effective for smaller breasts, this technique was insufficient for larger deformities, especially those with significant ptosis and redundancy. To address these issues, later approaches employed a transverse elliptical excision with repositioning of the nipple–areola complex (NAC), which is accomplished either by transposing it on a pedicle or by performing a full-thickness graft [9].

According to Gusenoff, the management of grade III pseudogynecomastia is the best by excision and free NAC graft [3]. Though the disadvantages of this technique are the affection of NAC sensation and the stuck on appearance of NAC, also not attacking the lateral chest redundancy. So in this technique, we tried to simultaneously treat the lateral chest with pseudogynecomastia in post-MWL patients also with preservation of sensation and natural appearance of NAC. This study aimed to asses long-term aesthetic outcomes of correction of severe pseudogynecomastia based on inferior pedicle from the patients and experts perspective.

## Patients and Methods

This is a prospective study which included 15 patients performed at Plastic, Burn and Maxillofacial Surgery Department, Ain Shams University Hospitals, Cairo, Egypt between January 2022 and January 2024. Study period was two years. All surgeries were done by the same surgeon.

It was approved by the Ethical Review Committee of the same institute. All patients signed an informed consent, and most interventions were done by the most experienced surgeon.

Patients with 18–35 years old, post-massive weight loss (MWL) following surgical or non-surgical interventions (MWL) with weight stability for 2 years after surgical weight loss and stable weight for at least 6 months were included, also pseudogynecomastia 3 according to Gusenoff classification. Individuals with previous gynecomastia surgery and congenital chest deformity were excluded. Preoperative photography and documentation of degree of ptosis were done.

### Surgical Technique

**Fig. 1:**
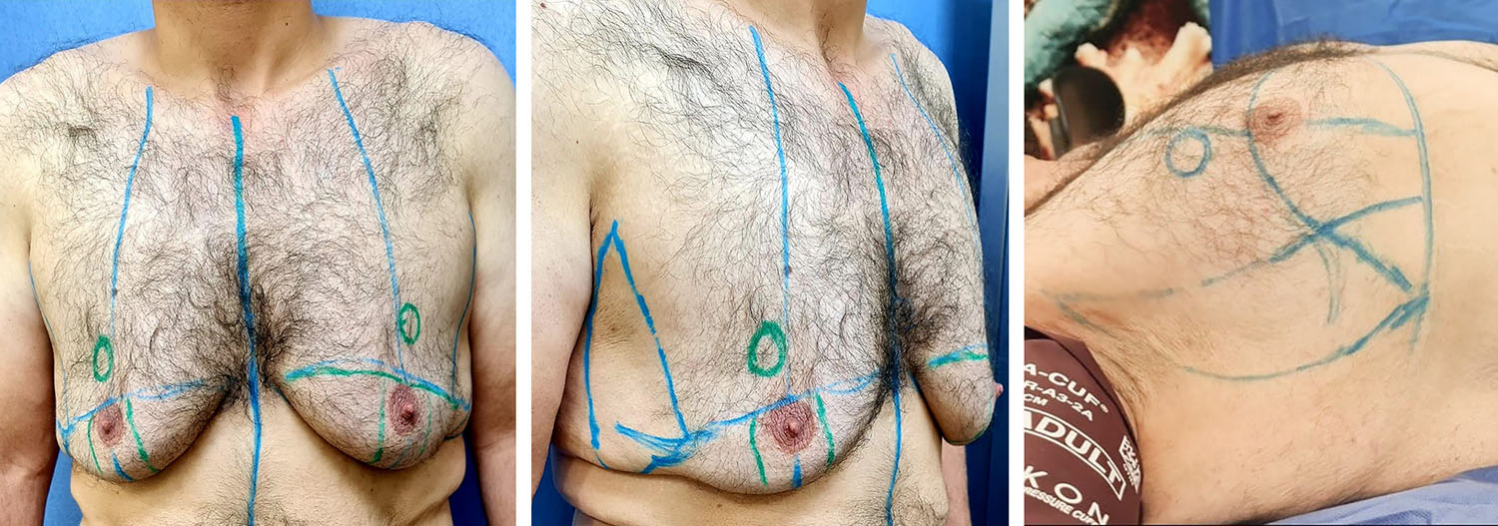
Preoperative markings. Left showing front view in standing position, middle showing oblique view in standing position and right showing lateral view in supine position

Markings were performed preoperatively on standing position (Fig. 1). The drawings delineated two ellipses:

- The first ellipse is a horizontal one with lower border that lies at the infra-mammary fold and the upper border which is an imaginary line determined by pinch test, and it always lies above the NAC.
- The second ellipse is a vertical one, drawn with the arms abducted, and its anterior border is just posterior to anterior axillary fold, and posterior border was detected by pinch test.

The intersection between horizontal and vertical ellipses lies at the infero-lateral angle of the pectoralis major muscle.

The inferior pedicle: The pedicle is centralized over the meridian with width at least 4 cm, extending from just above the areola down to the infra-mammary fold.

The new nipple–areola complex is marked just lateral to the breast meridian, 4 cm above the upper incision with diameter of the new nipple–areola complex as 2.5 cm.

Surgery was performed under general anesthesia. The patient was placed in the supine position with the arms abducted at 90. No liposuction was done. Then, it is proceeded with the de-epithelialization of the pedicle.

**Fig. 2:**
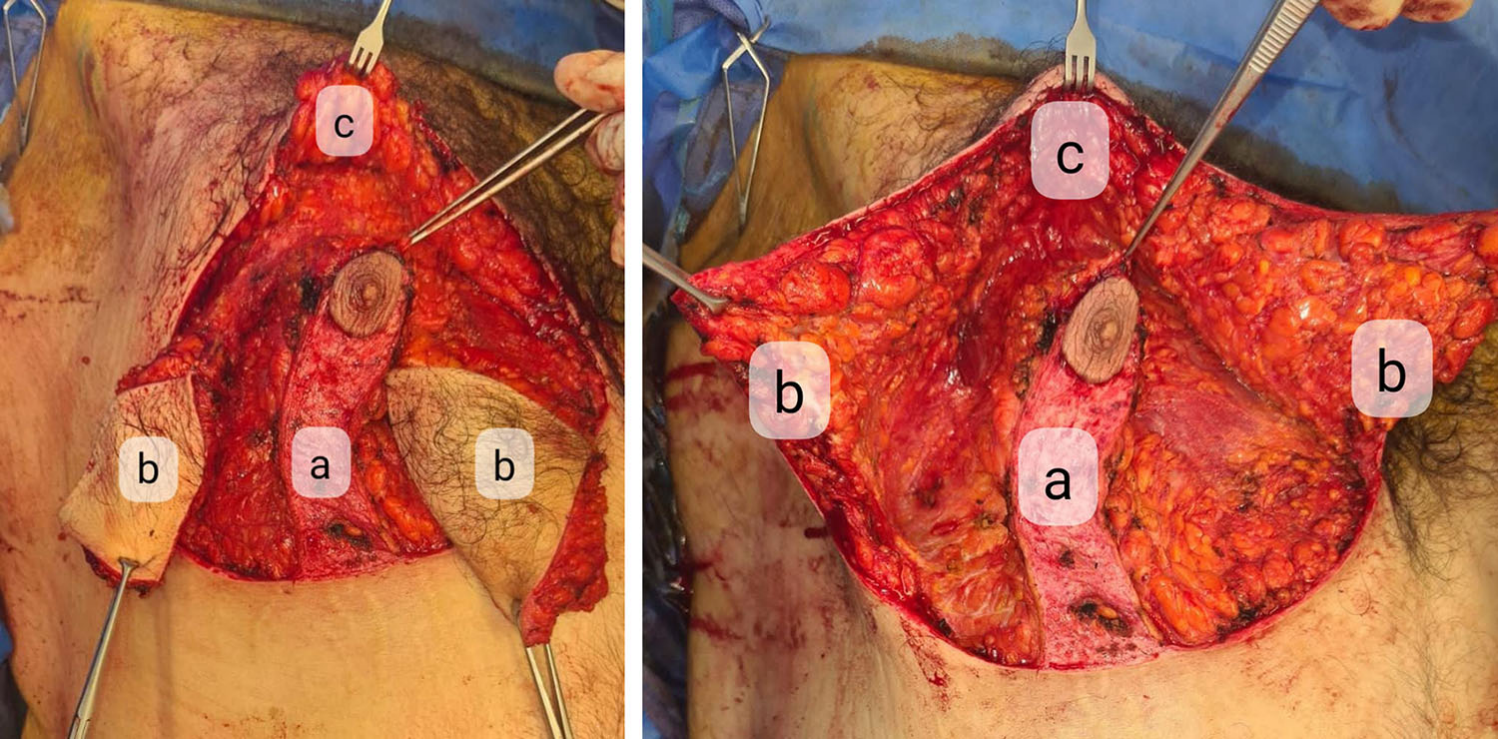
Intra-operative photographs. Left showing the inferior pedicle overlying the pectoral fascia, right showing the infra-mammary excess skin excision excess bounded by upper skin incision of horizontal ellipse, infra-mammary fold and the medial and lateral borders of the inferior pedicle. Where **a** represents the inferior pedicle, **b** represents the excess skin of the horizontal ellipse to be excised and **c** represents the upper chest flap

Procedure starts by incision over the lower line of the horizontal ellipse down to pectoral fascia and sparing the pedicle. Then, dissection proceeded over the fascia till upper border of the ellipse. After complete excision of the ellipse, the central 6 cm of the upper flap was debulked to help accommodation of the pedicle (Fig. 2).

**Fig. 3:**
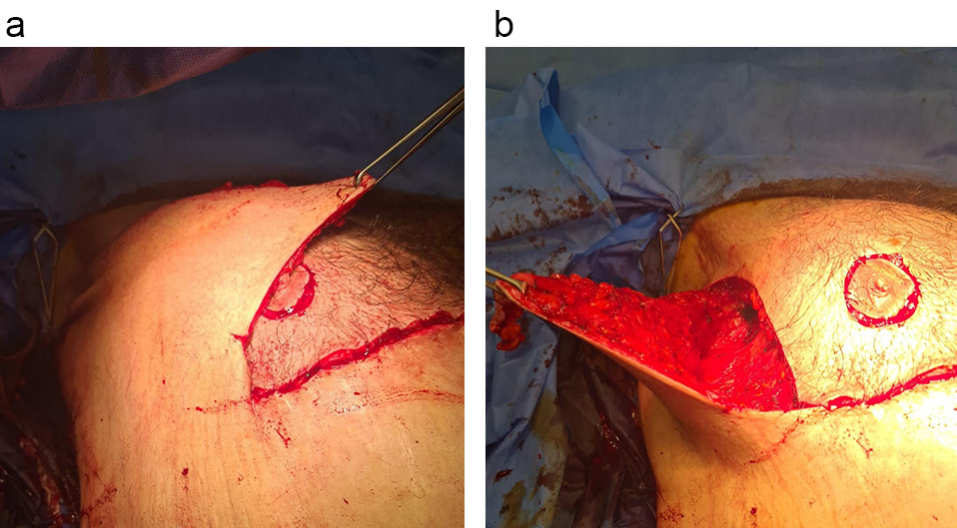
Excision of excess skin at lateral chest wall after transposition of nipple–areola complex with **a** showing the undermined lateral chest flap and **b** showing the excess skin needed to be excised

Three stay sutures were used to close the wound. A round full-thickness skin and subcutaneous block was excised at predetermined new location of the nipple–areola complex, and then the nipple–areola complex was transposed and secured by eight subcutaneous sutures with 3-0 PDS. Excision of the vertical ellipse started by incision over the anterior limb, and then the lateral flap and excess skin were excised. The lateral chest flap was suspended to the periosteum of underlying ribs and pectoral fascia by PDS 0 (Fig. 3).

**Fig. 4:**
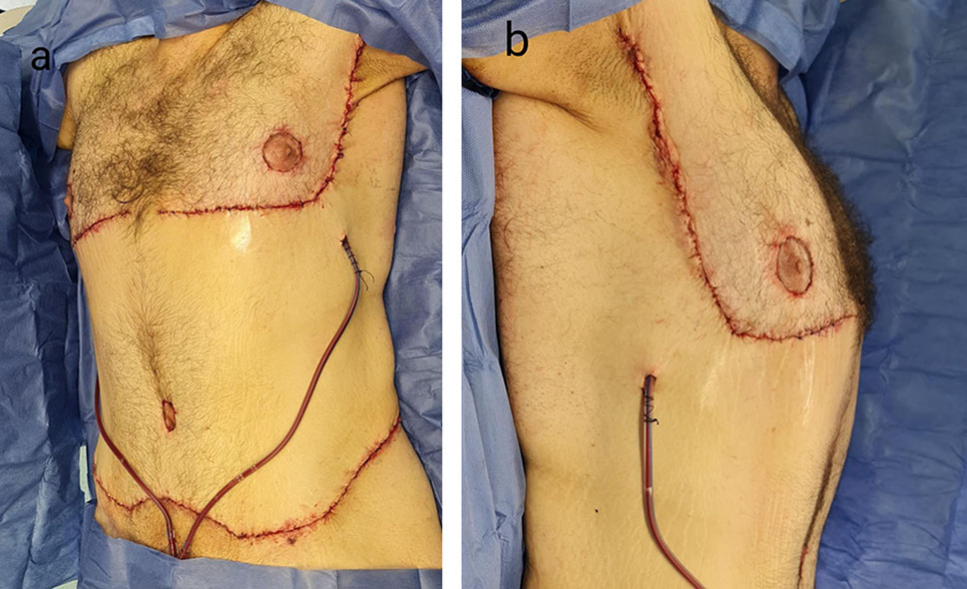
Immediate postoperative photographs with **a** showing the oblique view and **b** showing the lateral view

One suction drain was introduced at each side. Layered closure was done using subcutaneous sutures with 2-0 PDS and intradermal sutures with 3-0 monocryl (Fig. 4). Video of step wise approach is shown [video 1].

A compression garment was applied intraoperative. The garment was kept over for period of 2 months.

The patient was discharged the day after surgery, with the first dressing done after 1 week, and the drain was removed when output < 50 ml on 2 consecutive days with check for any complication like infection, hematoma, vascular compromise of NAC or skin flaps or any other complication.

**Fig. 5:**
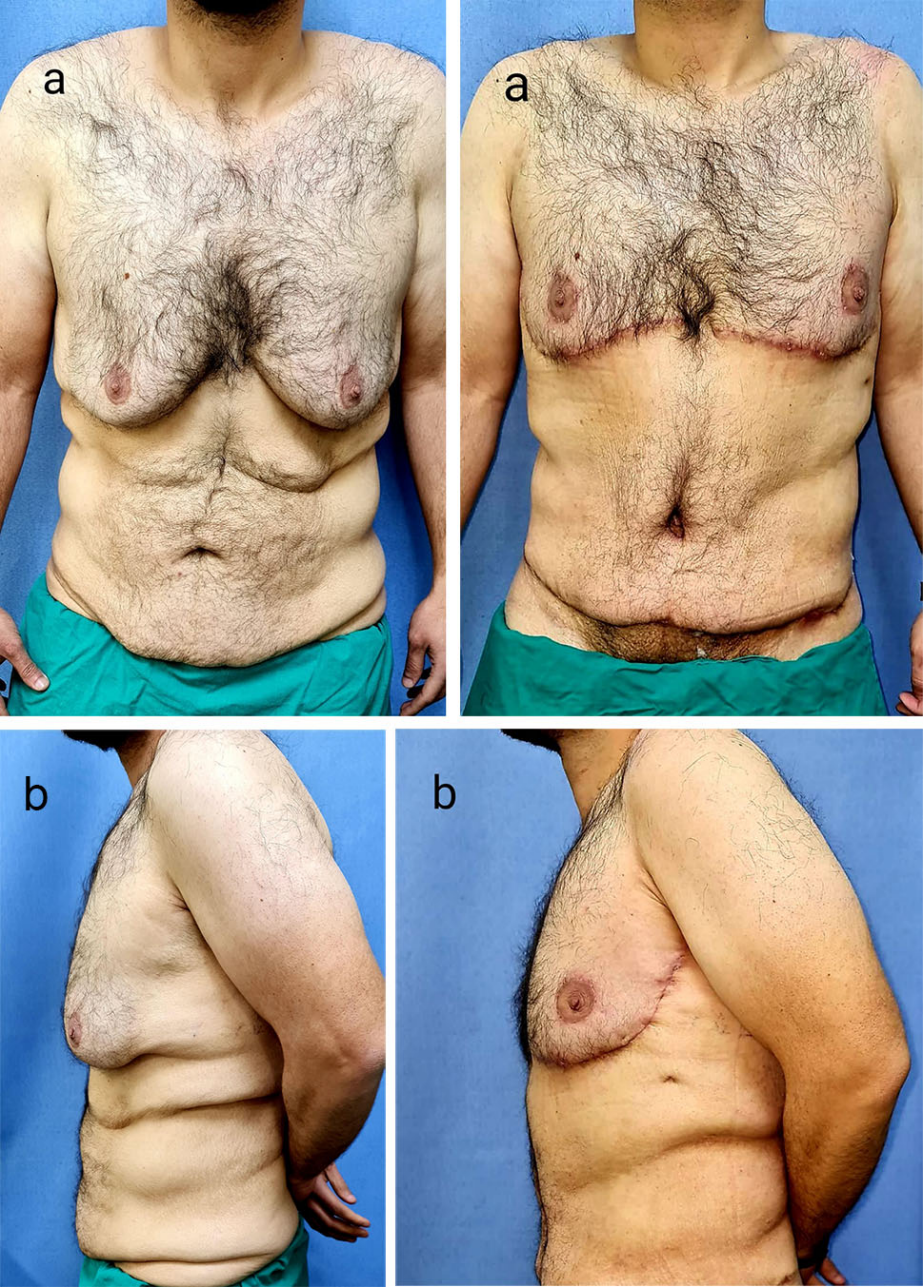
29-year-old patient. Preoperative photographs (Left: **a**: Frontal view, **b**: Left lateral view). Postoperative photographs at 1 month (Right: **a**: Frontal view, **b**: Left lateral view) showing improvement of chest contour, location of nipple–areola complex, lateral chest rolls and subcostal skin folds

Patient evaluation was done on 1, 3, 6 and 12 months (photographs were taken) (Fig. 5), and questionnaire for patient satisfaction was also done [3].

### Statistical Analysis

Statistical analysis was done by SPSS v20 (SPSS Inc., Chicago, Illinois, USA). Results were presented in the form of percentage for qualitative data and compared by using Chi-square test, mean and standard deviation in case of quantitative variables and compared by using independent t test. Statistical analysis for pre- and postoperative findings was performed using paired t test.

## Results

The ages ranged from 20 to 45 years (mean 32.67 years), The previous weight ranged from 130 to 200 with mean 158.8, the mean current weight was 87.5, the height ranged from 167 to 186 with mean 175.4, and the BMI ranged from 24 to 34.7 with mean. Regarding the method of weight loss, three (20.0%) cases had gastric bypass, three (20.0%) cases had life style modification and nine (60.0%) cases had sleeve gastrectomy. The time of weight loss ranged from 8 to 48 months (mean 20.07 months), and the duration of weight stability ranged from 6 to 24 months with mean 10.0 months. Table 1 shows the demographic data of all cases which had Grade III pseudogynecomastia according to Gusenoff’s classification. Tables 2 and 3 show history of the patients and their measurements respectively.

**Table 1:** Distribution of the studied cases according to age, previous weight, current weight, height, BMI, method of weight loss, time of weight loss and duration of weight stability

	No. = 15	
	Mean ± SD	Range
Age	32.67 ± 6.56	20–45
Previous weight	158.87 ± 16.96	130–200
Current Weight	87.53 ± 8.61	72–105
Height	175.40 ± 5.49	167–186
BMI	28.49 ± 2.89	24–34.7
Time of weight loss (Months)	20.07 ± 12.49	8–48
Duration of weight stability (Months)	10.00 ± 6.06	6–24
		N(%)
Method of weight loss	Gastric bypass	3 (20.0%)
	Life style modification	3 (20.0%)
	Sleeve gastrectomy	9 (60.0%)

BMI, Body mass index

**Table 2:** Distribution of the studied cases according to medical history, smoking, grades and concurrent procedures

	No.	%
Medical history		
Hypertension	3	20.0
DM	2	13.3
Smoker	8	53.3
Grade		
III	15	100.0

**Table 3:** Distribution of the studied cases according to sternal notch-to-nipple distance, nipple-to-infra-mammary fold, nipple to notch, areola diameter and chest circumference

	Pre	Post
*Sternal notch-to-nipple distance*		
Right	26.50 ± 1.59	18.93 ± 1.33
Left	26.37 ± 1.39	18.90 ± 1.39
*Nipple-to-infra-mammary fold*		
Right	7.70 ± 1.69	4.30 ± 1.16
Left	7.63 ± 1.73	4.24 ± 1.02
Nipple to nipple	22.30 ± 1.46	24.00 ± 2.17
*Areola diameter*		
Right	4.59 ± 0.81	2.71 ± 0.31
Left	4.67 ± 0.82	2.71 ± 0.32
*Chest circumference*	107.47 ± 8.82	105.47 ± 8.37

Data are presented as mean ± SD

**Fig. 6:**
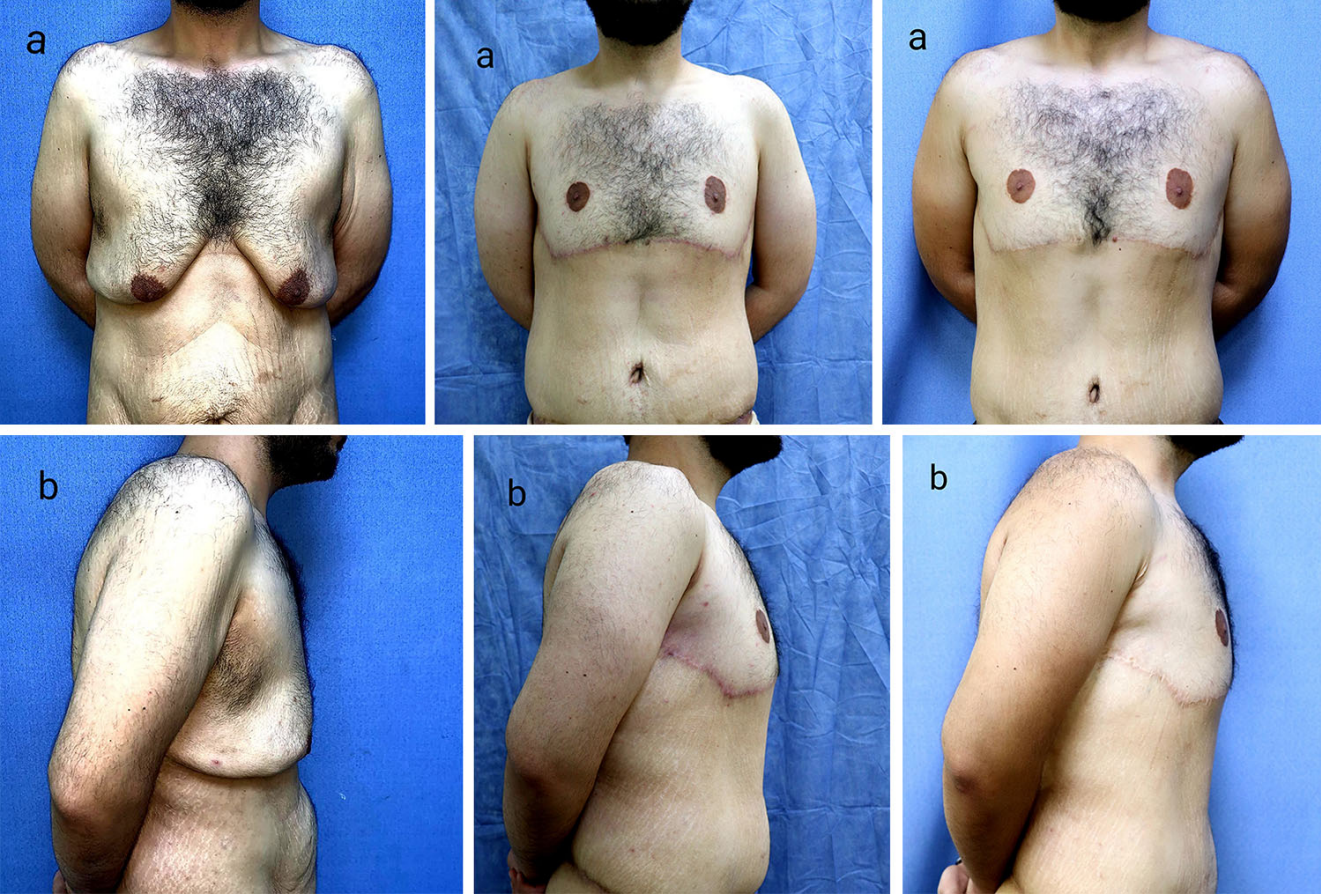
31-year-old patient. Preoperative photographs (Left: **a**: Frontal view, **b**: Left lateral view). Postoperative photographs at 3 months (middle: **a**: Frontal view, **b**: right lateral view). Postoperative photographs at 1 year (right: **a**: Frontal view, **b**: right lateral view) showing good chest contour with NAC asymmetry but the patient was satisfied and did not ask for revision

Regarding the complications, no major complications required readmission, four patients had minor complications (26.7%), one (6.7%) case had asymmetry of the NAC (Fig. 6), one (6.7%) case had minor hematoma that was managed by bedside aspiration, one (6.7%) case had seroma, and one (6.7%) case had partial wound dehiscence that were managed conservatively. Three out of four patients who had complications were smokers, but that is statistically insignificant due to small sample size. There were no infection or vascular compromise of the nipple–areola complex (Table 4). Regarding the scar assessment, the score according to Vancouver scar scale ranged from 2 to 6 (mean 3.13).

**Table 4:** Distribution of the studied cases according to complications and smoking status

Complications	No. = 15	Smoking status
Hematoma	1 (6.7%)	Smoker
Seroma	1 (6.7%)	Smoker
Asymmetry of the NAC	1 (6.7%)	Non-smoker
Wound dehiscence	1 (6.7%)	Smoker

Data are presented as mean ± SD or frequency (%)

**Fig. 7:**
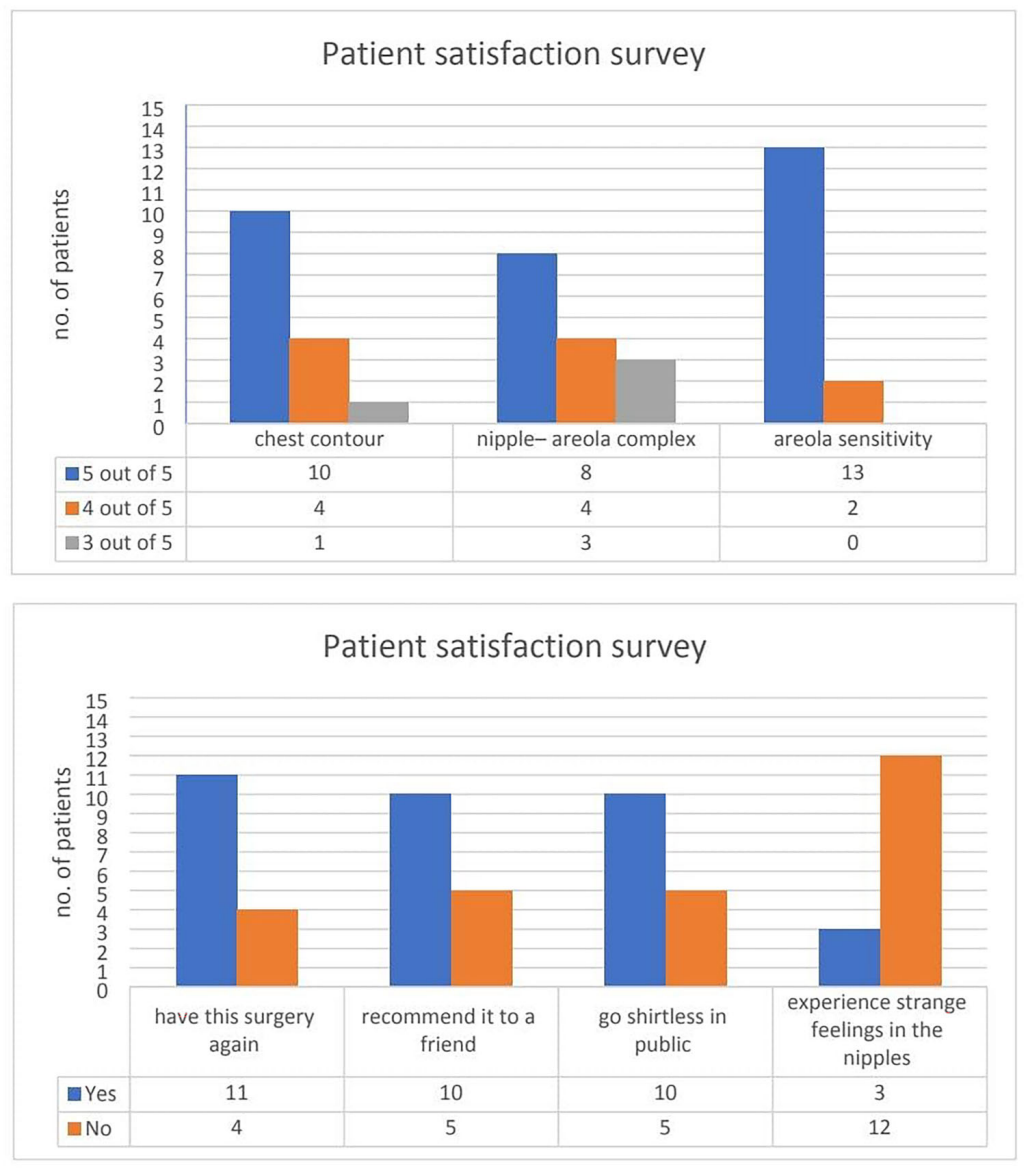
Results of patient satisfaction survey

Regarding the patient satisfaction survey [3], 11 (73.3%) cases would have this surgery again, ten (66.7%) would recommend it to a friend, ten (66.7) cases would go shirtless in public, and three (20%) cases had experienced strange feelings in the nipples. While the mean patient satisfaction rate for chest contour was 4.6., nipple–areola complex was 4.33. All cases reported regaining nipple–areola sensation (Table 5) (Fig. 7).

**Table 5:** Distribution of the studied cases according to patient satisfaction survey

Patient Satisfaction Survey	Out of 5
Chest contour	4.60 ± 0.63
Nipple–areola complex	4.33 ± 0.82
Areola sensitivity	4.87 ± 0.35
	No. = 15
Have this surgery again	11 (73.3%)
Recommend it to a friend	10 (66.7%)
Go shirtless in public	10 (66.7%)
Experience strange feelings in the nipples	3(20%)

Data are presented as mean ± SD or frequency (%)

## Discussion

For MWLP patients, the optimum body contouring technique should restore accepted chest contour with good aesthetics and position of NAC. These objectives cannot be done with MWLP without leaving a significant scar on the chest wall in order to remove all of the redundant, stretched skin from the chest, axilla, lateral chest rolls and upper abdomen [3].

This study presents the results of patients treated with the inferior pedicle technique for Grade III pseudogynecomastia post-weight loss. We initially do no liposuction, as in MWL patients the main issue is not excess fat but excess skin. In our experience, we could achieve good chest contour without liposuction, minimizing the risk of seroma formation and vascularity compromise of the upper chest flap. To achieve the best chest contour, we do debulking of the upper chest flap that corresponds the pedicle to avoid the bulge of the pedicle after insetting of the upper chest flap over it. To deal with the lateral chest, we extend the wound in fish tail manner just behind the anterior axillary fold to restore the masculine appearance of the male chest. Good nipple–areola complex aesthetics could also be achieved, with nipple–areola complex at 4 cm above the incision to maintain the vascularity of the skin bridge and lies lateral to the breast meridian. Also in good relation to the pectoralis major muscle about 1–2 cm from its inferior border and 2.5–3 cm from its lateral border, which is concomitant with recent studies [10–12], the ages ranged from 20 to 45 years (mean 32.67 years). The previous weight ranged from 130 to 200 with mean 158.87, the mean current weight was 87.5, and the BMI ranged from 24 to 34.7 with mean 28.4.

For the treatment of pseudogynecomastia, Gusenoff described a classification system and an algorithm for management. According to the algorithm, grade I pseudogynecomastia is treated by anterior chest liposuction and direct excision of the lateral roll, if present. Patients with higher grades have a horizontal elliptical excision and extension to the axilla to encompass the lateral roll, whereas grade II abnormalities underwent pedicled repair and grade III get free nipple grafts. He stated that the pedicled technique to have better NAC aesthetics as free grafts often has an unnatural, flat appearance, increased scarring and pigmentation changes. Pedicled reconstruction is more time-consuming and carries a greater risk of flap and nipple necrosis, and Gusenoff advises grafting in patients with grade III pseudogynecomastia because they think the substantial upper abdominal laxity associated with this condition could compromise the vascularity of the pedicle [3].

Regarding the complications, according to our study, one (6.7%) case had hematoma, one (6.7%) case had asymmetry of the NAC that did not require revision , one (6.7%) case had seroma, and one (6.7%) case had partial wound dehiscence that were managed conservatively . All were smokers except for the patient with asymmetry of the NAC. Complication rate was higher among smokers, as three out of four patients who had complications were smokers. But that is statistically insignificant due to small sample size. All smoker patients were advised to stop smoking 4 to 6 weeks before surgeries. There are various strategies to assist individuals in quitting, including nicotine replacement therapy, counseling, and support group [13]. If not compliant, they were informed about the higher risks of smoking and that was added to the informed consent.

Hardy in his study underwent liposuction to create a smooth transition from the superior chest to the inferior chest as well as soften the IMF crease and kept the NAC on wide dermal pedicle inferiorly based, and he kept the base of the random flap wide to maintain an appropriate blood supply; despite this, one patient had poor nipple perfusion intraoperatively and was converted to free nipple graft. Regarding the overall complication rate, four patients (28.6%) had complications with one patient with partial nipple necrosis, asymmetry of the NACs was seen in one patient, one patient had mild hyperpigmentation of the NAC and asymmetry of the infra-mammary folds, and one patient developed seroma [7].

In our study, regarding the patient satisfaction survey, 11 (73.3%) cases would have this surgery again, ten (66.7%) would recommend it to a friend, ten (66.7) cases would go shirtless in public, and three (20%) cases had experienced strange feelings in the nipples.

Thienot described the postero-inferior pedicle flap technique with pedicle base 6 cm also with liposuction. Parallel to our study, the overall rate of minor complications was 33% (*n *= 3). The overall patient satisfaction with Thienot was excellent, and the assessment score was 4 for chest contour that was 3.8 in the Gusenoff’s study, while the mean patient satisfaction rate in our study was 4.6. For nipple–areola complex, it was 4.1 with Thienot and 3.8 with Gusenoff, and in our study, it was 4.33. All patients would go shirtless in public, while with no patient lost their nipple sensitivity, one described dysesthesia. Except for one patient, all would undergo the surgery again and would recommend it to a friend [14]. Consistent with our results, Gusenoff observed great patient satisfaction after management of pseudogynecomastia following significant weight loss [3].

Another study that was done by Ibrahim using an inferior pedicle flap that was designed with a base-to length ratio of 1.5:1 is accompanied with liposuction. In this study, twelve patients (21.8%) experienced minor complications. Fifty-one patients (92.8%) reported being satisfied with their outcome at follow-up, as there were no major complications [15].

Austin in his study used Safelipo technique along with inferior pedicle for NAC, with length-to-width ratio (1:1). Progressive tension sutures were used with no drains. One patient had delayed wound healing (11.1%), and one patient (11.1%) had scar revision. According to all patients, NAC sensitivity was maintained. The aesthetic outcomes were good, and patients reported being satisfied with the results [16]. The reliability of the chest flap could be assessed by immediately checking vascularity of nipple–areola complex and later by sono-mammography [17].

Although our study provides a safe and reliable technique for management of pseudogynecomastia in post-MWL and shows promising outcomes regarding chest contour, aesthetics of NAC, rate of complications and patient satisfaction, the small sample size of this study limits the study’s findings.

## Conclusions

Patient satisfaction with male chest contouring for pseudogynecomastia following significant weight loss is excellent. In this study, we provide a reliable technique for management of pseudogynecomastia in post-MWL with good outcomes.

## Supplementary Information

Below is the link to the electronic supplementary material.

Supplementary file1 (MP4 64170 KB)
